# Dengue Hemorrhagic Fever in Infants: Research Opportunities Ignored

**DOI:** 10.3201/eid0812.020170

**Published:** 2002-12

**Authors:** Scott B. Halstead, Nguyen Trong Lan, Thein Thein Myint, Than Nu Shwe, Ananda Nisalak, Siripen Kalyanarooj, Suchitra Nimmannitya, Soegeng Soegijanto, David W. Vaughn, Timothy P. Endy

**Affiliations:** * Uniformed Services University of the Health Sciences, Bethesda, Maryland, USA; † Children’s Hospital No. 1, Ho Chi Minh City, Vietnam; ‡ Yangon Children’s Hospital, Yangon, Myanmar;; § Armed Forces Research Institute of the Medical Sciences, Bangkok, Thailand; ¶ Queen Sirikit National Institute of Child Health, Bangkok, Thailand; # Dr. Soetomo Hospital, Airlangga University Medical School, Surabaya, Indonesia

**Keywords:** dengue, dengue virus, viral hemorrhagic fevers, flavivirus, immunology, epidemiology, pathogenesis, infant, infectious diseases, feline infectious peritonitis virus

## Abstract

The age distribution of cases of dengue hemorrhagic fever and dengue shock syndrome (DHF/DSS) in infants under the age of 1 year are reported from Bangkok, Thailand, and for the first time for Ho Chi Minh City, Vietnam; Yangon, Myanmar; and Surabaya, Indonesia. The four dengue viruses were isolated from Thai infants, all of whom were having a primary dengue infection. Progress studying the immunologically distinct infant DHF/DSS has been limited; most contemporary research has centered on DHF/DSS accompanying secondary dengue infections. In designing research results obtained in studies on a congruent animal model, feline infectious peritonitis virus (FIPV) infections of kittens born to FIPV-immune queens should be considered. Research challenges presented by infant DHF/DSS are discussed.

 Since World War II, the four dengue viruses (formal name: *Dengue virus* [DENV]) have progressively spread geographically throughout the tropics, resulting in a global pandemic with tens of millions of infections annually, including several hundred thousand hospitalizations for dengue hemorrhagic fever (DHF) and dengue shock syndrome (DSS) (1). The size and spread of the dengue pandemic, the unpredictability of epidemic occurrences, and the circulation of virulent and nonvirulent strains make DHF/DSS a model for an emerging infectious disease.

Ample evidence suggests that DHF/DSS accompany secondary dengue infections in children older than 1 year (1–3). Less well-known are the epidemiologic and clinical studies that document an identical severe syndrome in infants during their first dengue infection (4,5). Ignoring these data, contemporary models of dengue immunopathogenesis focus on the sequential dengue viral infection phenomenon; such models suggest that severe disease results from amplified cytokine release caused by dengue infections occurring in the presence of T-cell memory (6). However, that model cannot explain DHF/DSS during a first dengue infection.

That dengue in infants is not often studied is understandable. Small subjects pose technical difficulties in obtaining samples required by research protocols, and human use protocols may be constraining. Yet infants represent 5% or more of all DHF/DSS patients (7). Uniquely, infants with DHF/DSS present an opportunity to obtain both the causative virus and the preinfection antibodies as research reagents in a hospital setting without recourse to a time-consuming and expensive prospective cohort study. The all-important preinfection antibodies can be collected from the mother, as her serum is a surrogate for cord blood (8).

Enhancement of infant infectious diseases by cord blood antibodies is not described for human infections other than dengue. However, such a phenomenon occurs naturally in infected kittens born to queens immune to feline infectious peritonitis virus (FIPV) (9–11). To refocus attention on the research opportunities afforded by this immunopathologic entity, we provide evidence that infants with DHF/DSS are regularly admitted to hospitals in four of the largest dengue-endemic countries. The age distribution of all these infant DHF/DSS patients is similar. Most of those studied serologically had had primary dengue infections. Because of FIPV’s congruence to infant dengue, a short literature review is provided on that animal model.

## Materials and Methods

### Patients

Data on infants, ages <12 months, hospitalized with a clinical diagnosis of DHF were obtained from four hospitals: Children’s Hospital No. 1, Ho Chi Minh City, Vietnam; the Queen Sirikit National Institute of Child Health, also referred to as Bangkok Children’s Hospital, Bangkok, Thailand; Children’s Hospital, Yangon, Myanmar; and the Department of Pediatrics, Dr. Soetomo Hospital, Surabaya, Indonesia. In this study, data for 4 consecutive years, either 1995–1998 or 1996–1999 were combined. Patients were under the routine care of one or more of the authors, each an experienced senior academic infectious diseases pediatrician. All diagnoses of DHF/DSS in infants conformed to World Health Organization case definitions. In Bangkok, serum samples from all infants and children hospitalized for DHF were sent for routine diagnostic study to the Virology Department, Armed Forces Research Institute of Medical Sciences (AFRIMS). For nearly 30 years, AFRIMS has provided such dengue diagnostic services to Bangkok Children’s Hospital. Similar routine diagnostic tests were provided for infants and children admitted to Children’s Hospital, Yangon, by the Virology Department, Department of Medical Research. Fiscal constraints limited the number of serologic tests performed. Individual data were disassociated from any identifiers and are presented here only in aggregate.

### Virus Isolation

As described, DENV isolations were attempted from acute-phase plasma or serum samples from Thai children by inoculation into C6/36 cells or intrathoracically in mosquitoes (*Toxorrhynchites splendens)* (12).

### Viral Identification

DENV was identified in C6/36 cells by an antigen-specific enzyme-linked immunosorbent assay (ELISA) with a panel of monoclonal antibodies against DENV (13).

### Serology

Plasma or serum samples were tested for serologic evidence of acute DENV infection by immunoglobulin (Ig) M and IgG ELISA, hemagglutination inhibition (HAI) assays, or both (14). For single specimens, 40 U of IgM to DENV was considered evidence of a DENV infection. A DENV IgM-to-IgG ratio >1.8 defined a primary infection. A ratio <1.8 defined a secondary DENV infection. With serial specimens, twofold increase in IgG to DENV with an absolute value of >100 U indicated a secondary infection in the absence of IgM to DENV of >40 U.

 In Bangkok, HAI antibody against DENV types 1–4 and *Japanese encephalitis virus* were measured in all sera (15). A fourfold increase was considered positive for acute flavivirus infection. The infection was diagnosed as primary if titers >1 week after onset of illness were <1:1,280 or as secondary if antibody titers were >1:1,280 (16).

## Results

**Figure 1:**
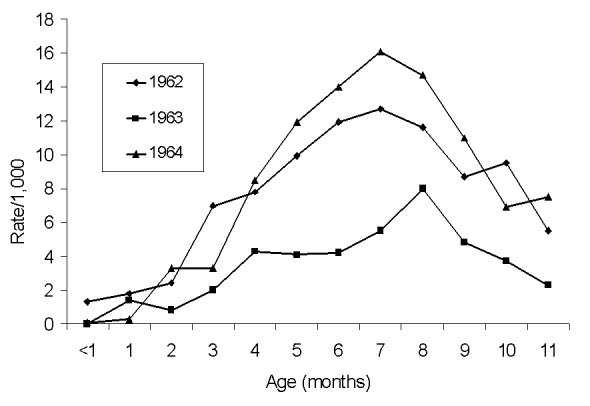
Age-specific hospitalization rates/1,000 infants with dengue hemorrhagic fever/dengue shock syndrome, Bangkok, Thailand, 1962–1964. Source: Halstead SB, et al. Am J Trop Med Hyg (17); cited with permission.

 Infants are at high risk for DHF/DSS. Figure 1 provides data from the only published study to estimate age-specific dengue hospitalization rates for the Bangkok metropolitan area. In 1964, 17/1,000 seven-month-old infants, more than 1% of the population that age, were hospitalized for DHF/DSS (17). This modal rate was two times higher than the 1964 modal hospitalization rate for children (age 4 years, data not shown) in Bangkok during the same year (17). In our present study, infant DHF/DSS constituted 4.9%, 4.6%, 5.0%, and 4.9% of 4,872; 14,053; 8,938; and 2,057 Thai, Vietnamese, Myanmar, and Indonesian infants and children hospitalized with DHF in 1995–1998, respectively.

**Figure 2:**
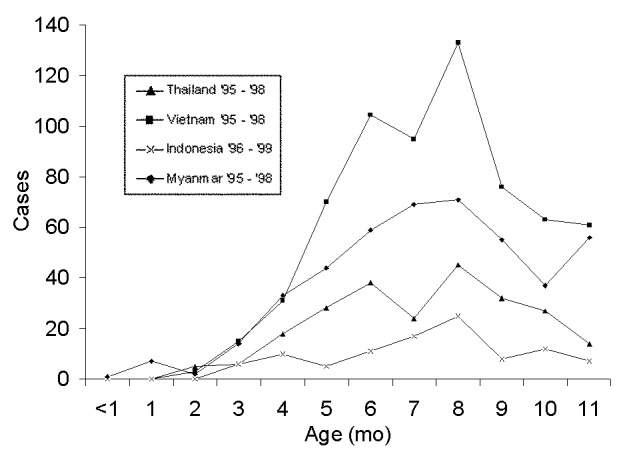
Month of age of infants hospitalized for dengue hemorrhagic fever/dengue shock syndrome at the Bangkok Children’s Hospital, 1995–1998 (Thailand), Children’s Hospital No.1, Ho Chi Minh City, 1995–1998 (Vietnam), Yangon Children’s Hospital, 1995–1998 (Myanmar) and Dr. Soetomo Hospital, Surabaya, 1996–1999 (Indonesia). Data are combined for the period shown.

 During a 4-year period, 237, 652, 449, and 101 infants with presumptive DHF/DSS were admitted to hospitals in Bangkok, Ho Chi Minh City, Yangon, and Surabaya. No significant differences in age distributions were observed year to year (data not shown). Data for 4 years were combined to smooth age distributions (Figure 2). Among infants hospitalized at Bangkok Children’s Hospital, the distribution of World Health Organization grades 1, 2, 3, and 4 was 16.4, 56.2, 23.9, and 3.0%, respectively. Only 2 of 220 infants whose serum samples were tested had a secondary-type antibody response, 97.5% had primary infections. DENV was isolated from 114 (nearly 50%) of Thai infants; DENV types 1, 2, 3, 4 were recovered from 34, 23, 56, and 1 infant, respectively. Nineteen Myanmar infants were serologically confirmed as having a recent primary infection; seven, mostly infants >10 months of age, had secondary-type dengue HAI-antibody responses.

**Figure 3:**
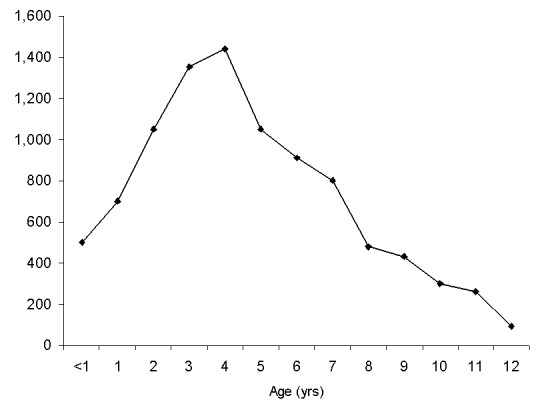
Year of age at hospitalization of children with dengue hemorrhagic fever/dengue shock syndrome, Yangon Children’s Hospital, Yangon, Myanmar, 1995–1998, combined.

The distribution by age of DHF/DSS infants in all four countries presents a similar pattern: few cases were observed in infants younger than 3 months, and the largest numbers observed were in infants 6–8 months old. Later in the first year, admissions declined nearly to baseline in Thailand and Indonesia. In Yangon, the decline in admissions reversed at age 10 months and increased. In Yangon, DHF hospitalizations continued to increase during the second year of life (Figure 3). In Ho Chi Minh City, cases in 11-month-olds declined, but not quite to the baseline.

**Figure 4:**
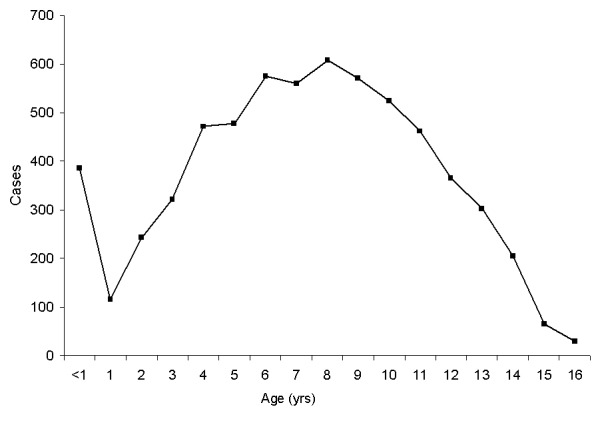
Year of age of children hospitalized for dengue hemorrhagic fever/dengue shock syndrome at Bangkok Children’s Hospital, Bangkok, Thailand, 1990–1999, combined.

Children hospitalized with DHF/DSS in Bangkok show the classical bimodal curve: relatively few cases in children 12–24 months of age and a modal age later in life, in this case at 8 years of age (Figure 4). By contrast, this bimodal distribution is not present in Yangon. DHF/DSS occurs commonly in 12- to 24-month-old children, and the modal age at admission is 4 years (Figure 3).

## Discussion

The patterns of age distribution of infant DHF/DSS were similar in four large Southeast Asian countries highly endemic for all four DENV serotypes. Nearly all infants in the large Thai and most in the smaller Myanmar groups had primary DENV antibody responses. The characteristic and unique age-specific hospitalization curves are consistent with published observations that describe infant DHF/DSS occurrence during primary DENV infections. Primary and secondary DHF infections are reconciled in a long-standing explanatory hypothesis linking severe disease to actively or passively acquired antibodies (4,8,18). In our study, infants constituted approximately 5% of total DHF/DSS patients, lower than the nearly 10% reported from Bangkok Children’s Hospital in the 1960s (7,17).

Differences were observed in the age distribution curves in Yangon and Ho Chi Minh City compared with Bangkok and Surabaya. In Yangon, the curve declined at 8 months but rose again at age 10 months, and no dip in cases occurred in 1-year-old children (Figures 2 and 3). In Ho Chi Minh City, infant cases declined at the end of the first year of life, but not to the baseline; by contrast, in Thailand and Indonesia by the end of the first year of life, the curve approached the baseline. These phenomena may be explained by differences in average annual rates of dengue infection. In Yangon, the modal age of hospitalization for DHF/DSS for children is 4 years (Figure 3), while in Bangkok it is 8 years (Figure 4). In Bangkok, DHF/DSS is rarely seen in 12- to 24-month-olds, signifying that second infections are usually delayed until after a child has lived through two dengue transmission periods (Figure 4). Among serologically studied infant DHF/DSS patients in Yangon, several ages 10 months and older had secondary dengue infections. These observations are consistent with high average annual rates of dengue infection in Yangon and lower infection rates in Bangkok. These relationships have been modeled mathematically (19).

**Figure 5:**
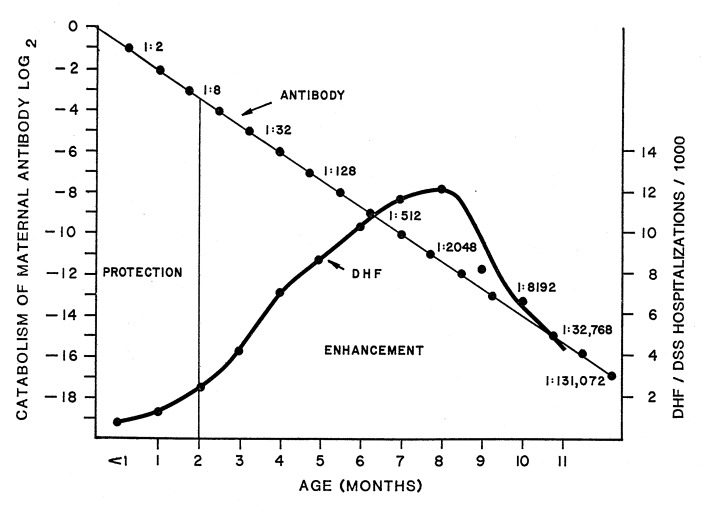
Relationship between the age distributions of infants hospitalized for dengue hemorrhagic fever/dengue shock syndrome (DHF/DSS) and the protective and infection-enhancing effects of maternal dengue antibodies. Shown are mean age specific hospitalization rate/1,000 for Bangkok and Thonburi, 1962–1964 (see Figure 1). At birth, antibodies are at protective concentrations. With the passage of time, maternal immunoglobulin G antibodies are catabolized to concentrations that result in antibody-dependent enhancement (ADE) of infections. By the end of the first year of life, ADE antibodies are catabolized to concentrations below the ADE threshold, and DHF/DSS cases disappear.

Since infant DHF/DSS was first reported in 1970 (4), only a single research study has been undertaken on this group (8). This study included 13 Bangkok infants, all with primary DENV-2 infections, who were admitted to hospital at different ages in the first year of life. During hospitalization, mother’s blood was taken and tested as a surrogate for cord blood at birth. An analysis of dengue-neutralizing antibodies showed that every mother in the study had had two or more previous DENV infections (8). All infants acquired DHF/DSS during the short window of time when maternal DENV-2 neutralizing antibodies had degraded to a titer of approximately 1:10. Maternal sera enhanced DENV-2 at high dilutions. These data provide a logical explanation for the observed age distribution of infant DHF/DSS. At birth, maternal antibodies protect infants from dengue infection. As IgG antibodies are catabolized, a period of risk to enhanced infection ensues, followed in turn by the loss of enhancing antibodies and a corresponding decline in risk for DHF/DSS (Figure 5).

Data from studies on infants as well as prospective cohort studies on children demonstrate that the waning or absence of heterotypic neutralizing antibodies permits enhanced infections to occur. Infection enhancement occurs at lower antibody concentrations than neutralization (8,20).

Immunopathogenesis mechanisms have been more extensively studied in a remarkably similar viral infection of cats, FIPV, a highly fatal coronavirus disease of domestic and exotic cats (9–11). Most cats naturally exposed as adults to FIPV develop antibody titers without showing clinical signs. Lesions in sick cats are believed to result from immunologically mediated responses (9,10,21). Kittens receiving apparently competent neutralizing antibodies to FIPV, transferred in colostrum from immune queens, develop a fatal disease a few days after infection with wild-type virus (22). Passive transfer of antibody by other routes produces the same result (22,23). This phenomenon is called the early death syndrome. FIPV in antibody-negative kittens occurs less reliably and is delayed for several weeks until animals develop their own antibody response to the virus. FIPV in kittens is characterized by thrombocytopenia and elevated ALT, AST, and serum bilirubin (22).

The coronaviruses, pathogens of mammals and birds, are a large family of enveloped RNA viruses with a nonsegmented, positive-stranded genome that is 27–32 kb in length (24). One of the most intriguing aspects of coronavirus replication is the occurrence of high-frequency homologous RNA recombination (25). Together with porcine *Transmissible gastroenteritis virus* (TGEV), *canine coronavirus*, and human coronavirus 229E (HCV), the feline coronaviruses form a separate cluster within the genus *Coronavirus,* including Feline enteric coronavirus, and FIPV (26). Coronavirus virions possess three structural proteins, a large spike glycoprotein (S), a small integral membrane glycoprotein (M), and a nucleocapsid protein (N) (24). These proteins are analogous to the envelope (E), M, and nucleocapsid (C) proteins of the flaviviruses. The feline coronaviruses can be divided into two serotypes, I and II, on the basis of cross-reactivity to *canine coronavirus* in virus neutralization assays (26). Type I viruses grow poorly in tissue cultures and show virtually no neutralization with anti–*canine coronavirus* sera (27). Type II viruses grow readily in vitro (28). Analysis of gene structure suggests that type II viruses are derived from recombination of type I feline coronavirus and *canine coronavirus* (26). The two serotypes circulate as two pathotypes, the avirulent enteric viruses and the virulent FIPV (26). High-frequency mutations may help coronaviruses escape neutralization and promote infection enhancement in Fc receptor–bearing cells.

Antibody-dependent enhancement of FIPV has been demonstrated in vitro in feline macrophages as well as in stable human and mouse macrophage cell lines (29). More cells are infected in the presence compared with the absence of antibody; the rates of viral entry and viral replication are similar under both conditions (30). Coronaviruses appear to enter mononuclear phagocytes by means of the plasma membrane without marked involvement of phagocytic or endosomal pathways (28). Some researchers have surmised that, as with DENV, when antibody-virus complexes attach to Fc-receptors, viruses are brought close to cell surfaces, where they enter the cells by normal mechanisms (31,32). Enhancement is mediated by clusters of epitopes on the S protein (33,34). Results with FIPV suggest that feline IgG2a antibodies mediate both neutralization and enhancement (33). Antibody-dependent enhancement in FIPV demonstrates a bell-shaped curve with increasing dilutions; maximal enhancement occurs at subneutralizing titers (34).

Antibody-dependent enhancement is believed to be the cause of vaccine failure after immunization with live (35,36) or recombinant (37) vaccines. Inoculation of cats with a recombinant vaccinia virus expressing the S protein FIPV 79-1146 sensitized cats and led to accelerated disease after FIPV challenge (37), while inoculation with recombinant vaccinia viruses expressing the M or N proteins did not (38). Immunization with vaccines made from other members of the feline coronavirus group, TGEV or *canine coronavirus,* also sensitizes cats to early death syndrome (39).

*Flaviviridae* do not appear to be subject to as high rates of homologous recombination as are the *Coronaviridae*; nonetheless, during evolutionary history four dengue serotypes have emerged. A phenomenon reminiscent of the feline coronaviruses is the evidence that DENV also circulate as two biotypes: DENV-2 American genotype does not cause DHF/DSS, while DENV-2 SE Asian genotype does (40). As with feline coronaviruses, the severity of disease with the two DENV biotypes may be regulated by cross-reactive antibodies (41). Focused research on viral-antibody interactions at the structural level might clarify early pathogenesis events in DHF/DSS. Infants may provide an accessible and inexpensive model to study mechanisms controlling the severity of dengue infections. Workers actively involved in developing dengue vaccines may benefit from lessons learned in the FIPV model.

## References

[1] Halstead SB. Pathogenesis of dengue: challenges to molecular biology. Science. 1988;239:476–81. 10.1126/science.3277268 3277268

[2] Vaughn DW, Green S, Kalayanarooj S, Innis BL, Nimmannitya S, Suntayakorn S, Dengue viremia titer, antibody response pattern, and virus serotype correlate with disease severity. J Infect Dis. 2000;181:2–9. 10.1086/315215 10608744

[3] Ngo NTCX, Kneen R, Wills B, Nguyen VMN, Nguyen TQ, Chu VT, Acute management of dengue shock syndrome: a randomized double-blind comparison of four intravenous fluid regimens in the first hour. Clin Infect Dis. 2001;32:204–13. 10.1086/318479 11170909

[4] Halstead SB, Nimmannitya S, Cohen SN. Observations related to pathogenesis of dengue hemorrhagic fever. Iv. Relation of disease severity to antibody response and virus recovered. Yale J Biol Med. 1970;42:311–28. 5419206 PMC2591704

[5] Halstead SB, Nimmannitya S, Yamarat C, Russell PK. Hemorrhagic fever in Thailand; recent knowledge regarding etiology. Jpn J Med Sci Biol. 1967;20:96–103. 5301574

[6] Rothman AL, Ennis FA. Immunopathogenesis of dengue hemorrhagic fever. Virology. 1999;257:1–6. 10.1006/viro.1999.9656 10208914

[7] Halstead SB. Immunological parameters of Togavirus disease syndromes. In: Schlesinger RW, editor. The Togaviruses, biology, structure, replication. New York: Academic Press; 1980. p. 107–73.

[8] Kliks SC, Nimmannitya S, Nisalak A, Burke DS. Evidence that maternal dengue antibodies are important in the development of dengue hemorrhagic fever in infants. Am J Trop Med Hyg. 1988;38:411–9. 3354774 10.4269/ajtmh.1988.38.411

[9] Horzinek MC, Osterhaus AD. Feline infectious peritonitis: a coronavirus disease of cats. Small Anim Pract. 1978;19:623–30. 10.1111/j.1748-5827.1978.tb05551.x 213654

[10] Horzinek MC, Osterhaus AD. The virology and pathogenesis of feline infectious peritonitis: brief review. Arch Virol. 1979;59:1–15. 10.1007/BF01317889 218528 PMC7087126

[11] Weiss RC, Scott FW. Feline infectious peritonitis. In: Kirk RW, editor. Current veterinary therapy. Philadelphia: W.B. Saunders; 1980. p. 1288–92.

[12] Vaughn DW, Green S, Kalayanrooj S, Innis BL, Nimmannitya S, Suntayakorn S, Dengue in the early febrile phase: viremia and antibody responses. J Infect Dis. 1997;176:322–30. 10.1086/514048 9237696

[13] Kuno G, Gomez I, Gubler DJ. An ELISA procedure for the diagnosis of dengue infections. J Virol Methods. 1991;33:101–13. 10.1016/0166-0934(91)90011-N 1939502

[14] Innis BL, Nisalak A, Nimmannitya S, Kusalerdchariya S, Chongswasdi V, Suntayakorn S, An enzyme-linked immunosorbent assay to characterize dengue infections where dengue and Japanese encephalitis co-circulate. Am J Trop Med Hyg. 1989;40:418–27. 2540664 10.4269/ajtmh.1989.40.418

[15] Clarke DH, Casals J. Techniques for hemagglutination and hemagglutination inhibition with arthropod-borne viruses. Am J Trop Med Hyg. 1958;7:561–73. 13571577 10.4269/ajtmh.1958.7.561

[16] World Health Organization. Dengue haemorrhagic fever: diagnosis, treatment, prevention and control. 2nd edition. Geneva: The Organization; 1997.

[17] Halstead SB, Scanlon J, Umpaivit P, Udomsakdi S. Dengue and chikungunya virus infection in man in Thailand, 1962–1964: IV. Epidemiologic studies in the Bangkok metropolitan area. Am J Trop Med Hyg. 1969;18:997–1021. 4390977 10.4269/ajtmh.1969.18.997

[18] Halstead SB. Observations related to pathogenesis of dengue hemorrhagic fever. VI. Hypotheses and discussion. Yale J Biol Med. 1970;42:350–62. 5419208 PMC2591710

[19] Fischer DB, Halstead SB. Observations related to pathogenesis of dengue hemorrhagic fever. V. Examination of age-specific sequential infection rates using a mathematical model. Yale J Biol Med. 1970;42:329–49. 5419207 PMC2591712

[20] Kliks SC, Nisalak A, Brandt WE, Wahl L, Burke DS. Antibody-dependent enhancement of dengue virus growth in human monocytes as a risk factor for dengue hemorrhagic fever. Am J Trop Med Hyg. 1989;40:444–51. 2712199 10.4269/ajtmh.1989.40.444

[21] Weiss RC, Scott FW. Pathogenesis of feline infectious peritonitis: nature and development of viremia. Am J Vet Res. 1981;42:382–90. 6267961

[22] Weiss RC, Scott FW. Antibody-mediated enhancement of disease in feline infectious peritonitis: comparisons with dengue hemorrhagic fever. Comp Immunol Microbiol Infect Dis. 1981;4:175–88. 10.1016/0147-9571(81)90003-5 6754243 PMC7134169

[23] Pederson NC, Boyle JF. Immunologic phenomena in the effusive form of feline infectious peritonitis. Am J Vet Res. 1980;41:868–76. 6254400

[24] Siddell SG. The *Coronaviridae.* In: Siddell SG, editor. The *Coronaviridae*. New York: Plenum Press;1995.

[25] Lai MMC. Recombination in large RNA viruses: coronaviruses. Semin Virol. 1996;7:381–8. 10.1006/smvy.1996.0046 PMC7172158 38620226

[26] Herrewegh AA, Smeenk I, Horzinek MC, Rottier PJ, de Groot RJ. Feline coronavirus type II strains 79-1683 and 79-1146 originate from a double recombination between feline coronavirus type I and canine coronavirus. J Virol. 1998;72:4508–14. 9557750 10.1128/jvi.72.5.4508-4514.1998 PMC109693

[27] Hohdatsu T, Tatekawa T, Koyama H. Enhancement of feline infectious peritonitis virus type I infection in cell cultures using low-speed centrifugation. J Virol Methods. 1995;51:357–62. 10.1016/0166-0934(94)00119-2 7738156 PMC7119760

[28] Olsen CW. A review of feline infectious peritonitis virus: molecular biology, immunopathogenesis, clinical aspects, and vaccination. Vet Microbiol. 1993;36:1–37. 10.1016/0378-1135(93)90126-R 8236772 PMC7117146

[29] Hohdatsu T, Tokunaga J, Koyama H. The role of IgG subclass of mouse monoclonal antibodies in antibody-dependent enhancement of feline infectious peritonitis virus infection of feline macrophages. Arch Virol. 1994;139:273–85. 10.1007/BF01310791 7832635 PMC7087006

[30] Olsen CW, Corapi WV, Jacobson RH, Simkins RA, Saif LJ, Scott FW. Identification of antigenic sites mediating antibody-dependent enhancement of feline infectious peritonitis virus infectivity. J Gen Virol. 1993;74:745–9. 10.1099/0022-1317-74-4-745 7682252

[31] Gollins SW, Porterfield JS. Flavivirus infection enhancement in macrophages: an electron microscopic study of viral cellular entry. J Gen Virol. 1985;66:1969–82. 10.1099/0022-1317-66-9-1969 4031825

[32] Mady BJ, Erbe DV, Kurane I, Fanger MW, Ennis FA. Antibody-dependent enhancement of dengue virus infection mediated by bispecific antibodies against cell surface molecules other than Fc-gamma receptor. J Immunol. 1991;147:3139–44. 1680925

[33] Corapi WV, Olsen CW, Scott FW. Monoclonal antibody analysis of neutralization and antibody-dependent enhancement of feline infectious peritonitis virus. J Virol. 1992;66:6695–705. 1383568 10.1128/jvi.66.11.6695-6705.1992 PMC240165

[34] Olsen CW, Corapi WV, Ngichabe CK, Baines JD, Scott FW. Monoclonal antibodies to the spike protein of feline infectious peritonitis virus mediate antibody-dependent enhancement of infection of feline macrophages. J Virol. 1992;66:956–65. 1309922 10.1128/jvi.66.2.956-965.1992 PMC240797

[35] Pedersen NC. Animal virus infections that defy vaccination: equine infectious anemia, caprine arthritis-encephalitis, maedi-visna, and feline infectious peritonitis. Adv Vet Sci Comp Med. 1989;33:413–28. 2539002 10.1016/B978-0-12-039233-9.50017-2 PMC7149982

[36] Pedersen NC, Black JW. Attempted immunization of cats against feline infectious peritonitis, using avirulent live virus or sublethal amounts of virulent virus. Am J Vet Res. 1983;44:229–34. 6299143

[37] Vennema H, DeGroot RJ, Harbour DA, Dalderup M, Gruffydd-Jones T, Horzinek MC, Early death after feline infectious peritonitis challenge due to recombinant vaccinia virus immunization. J Virol. 1990;64:1407–9. 2154621 10.1128/jvi.64.3.1407-1409.1990 PMC249267

[38] Vennema H, DeGroot RJ, Harbour DA, Horzinek M, Spaan WJM. Primary structure of the membrane and nucleocapsid protein genes of feline infectious peritonitis virus and immunogenicity of recombinant vaccine viruses in kittens. Virology. 1991;181:327–35. 10.1016/0042-6822(91)90499-2 1847259 PMC7130817

[39] Chalmers WSK, Horsburgh BC, Baxendale W, Brown TDK. Enhancement of FIP in cats immunize with vaccina virus recombinants expressing CCV and TGEV spike glycoproteins. In: Laude H, Vautherot JF, editors. Coronoviruses. New York: Plenum Press; 1994. p. 359–64.

[40] Watts DM, Porter KR, Putvatana P, Vasquez B, Calampa C, Hayes CG, Failure of secondary infection with American genotype dengue 2 to cause dengue haemorrhagic fever [see comments]. Lancet. 1999;354:1431–4. 10.1016/S0140-6736(99)04015-5 10543670

[41] Kochel TJ, Watts DM, Halstead SB, Hayes CG, Espinosa A, Felices V, Neutralization of American genotype dengue 2 viral infection by dengue l antibodies may have prevented dengue hemorrhagic fever in Iquitos, Peru. Lancet. 2002;360:310–2. 10.1016/S0140-6736(02)09522-3 12147378

